# The Feasibility and Accuracy of Sentinel Lymph Node Biopsy in Initially Clinically Node-Negative Breast Cancer after Neoadjuvant Chemotherapy: A Systematic Review and Meta-Analysis

**DOI:** 10.1371/journal.pone.0162605

**Published:** 2016-09-08

**Authors:** Chong Geng, Xiao Chen, Xiaohua Pan, Jiyu Li

**Affiliations:** Department of Breast and Thyroid Surgery, Shandong Provincial Hospital Affiliated to Shandong University, Jinan, Shandong Province, China; Fondazione IRCCS Istituto Nazionale dei Tumori, ITALY

## Abstract

**Background:**

With the increased use of neoadjuvant chemotherapy (NAC) in breast cancer, the timing of sentinel lymph node biopsy (SLNB) has become increasingly important. In this study, we aimed to evaluate the feasibility and accuracy of SLNB for initially clinically node-negative breast cancer after NAC by conducting a systematic review and meta-analysis.

**Methods:**

We searched PubMed, Embase, and the Cochrane Library from January 1, 1993 to November 30, 2015 for studies on initially clinically node-negative breast cancer patients who underwent SLNB after NAC followed by axillary lymph node dissection (ALND).

**Results:**

A total of 1,456 patients from 16 studies were included in this review. The pooled identification rate (IR) for SLNB was 96% [95% confidence interval (CI): 95%-97%], and the false negative rate (FNR) was 6% (95% CI: 3%-8%). The pooled sensitivity, negative predictive value (NPV) and accuracy rate (AR) were 94% (95% CI: 92%-97%, I^2^ = 27.5%), 98% (95% CI: 98%-99%, I^2^ = 42.7%) and 99% (95% CI: 99%-100%, I^2^ = 32.6%), respectively. In the subgroup analysis, no significant differences were found in either the IR of an SLNB when different mapping methods were used (P = 0.180) or in the FNR between studies with and without immunohistochemistry (IHC) staining (P = 0.241).

**Conclusion:**

Based on current evidence, SLNB is technically feasible and accurate enough for axillary staging in initially clinically node-negative breast cancer patients after NAC.

## Introduction

Although molecular mechanisms increasingly contribute to our understanding of breast cancer, the status of the axilla remains the most important prognostic factor for breast cancer patients [1–3]. Evaluating the status of the axilla in invasive breast cancer is important for both the prognosis and adjuvant treatment recommendations. Axillary lymph node dissection (ALND) has been a standard technique for axillary staging of breast cancer for over 100 years; however, the complications of ALND, which include lymphedema, axillary web syndrome, numbness, pain, and restriction of shoulder range of motion, decrease patients' quality of life greatly [4–6]. Sentinel lymph node biopsy (SLNB), a minimally invasive procedure, was introduced in the early 1990s [7]. SLNB showed high accuracy in determining the status of the axilla and led to less morbidity compared with ALND [8, 9]. SLNB has been studied extensively and recommended as an alternative to traditional ALND for axillary status staging in patients with early breast cancer [9–11].

Neoadjuvant chemotherapy (NAC) was initially introduced in the 1970s for the treatment of patients with locally advanced breast cancer [12]. Although compared with adjuvant chemotherapy, NAC cannot improve patients’ disease-free and overall survival [13, 14], it offers some potential advantages: It provides an additional opportunity for breast-conserving therapy [15, 16]; it enables the *in vivo* assessment of sensitivity to chemotherapy [17]; and it presents an opportunity to evaluate the effects of newly developed agents [18]. The role of NAC has changed considerably in recent decades. As a result, the indications for NAC have been extended to select patients with early breast cancer [15, 16, 19].

Despite the increasing use of both NAC and SLNB, the feasibility of SLNB after NAC remains controversial. It is possible that tumor eradication after NAC could alter lymphatic drainage via the fibrosis of lymphatic channels and lead to a decreased identification rate and an increased false negative rate in SLNB [20, 21]. At present, ALND is the standard treatment for the axilla following NAC.

For patients with clinically node-negative breast cancer before NAC, the timing of SLNB is controversial. According to guidelines of the American Society of Clinical Oncology (ASCO), breast cancer patients with clinically negative axillary nodes are candidates for SLNB for axillary staging [22]. To avoid the potential influence of NAC, SLNB is usually performed before NAC in this population of patients; however, there are also obvious advantages to implementing SLNB after NAC. If SLNB after NAC in initially clinically node-negative patients can achieve outcomes comparable to those of patients who did not undergo NAC, patients may only need a single surgery for both the breast and axilla instead of two separate operative procedures.

Several studies have assessed the feasibility and accuracy of SLNB in initially clinically node-negative breast cancer patients after NAC. The patient selection criteria, mapping methods and histological examinations vary across individual studies. Hence, we performed the present meta-analysis to collect data for evaluation purposes. To the best of our knowledge, this is the first meta-analysis to address the clinical question mentioned above.

## Methods

### Literature search strategy

**Fig 1:**
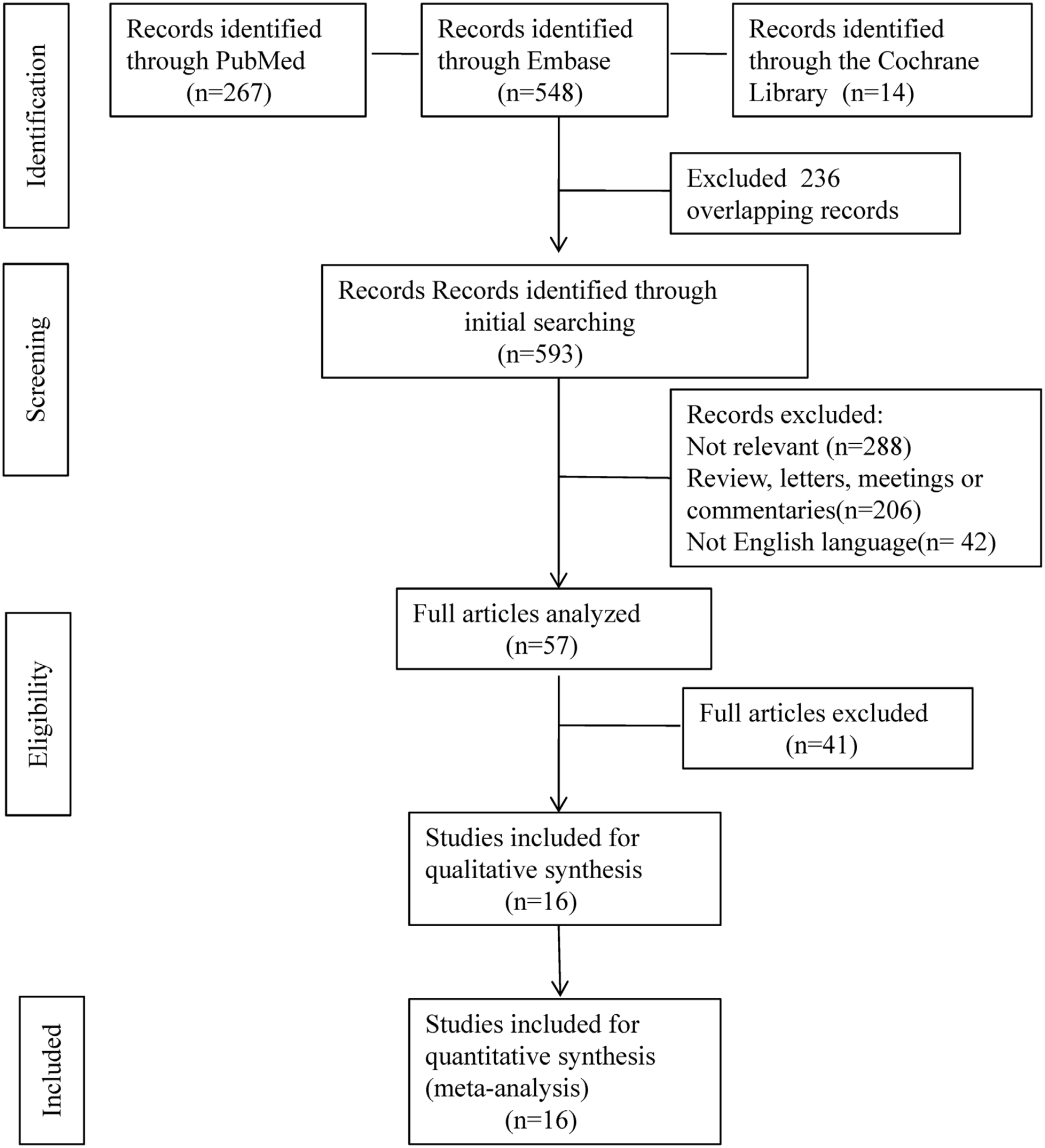
Flow diagram of literature search and individual studies identified for this systematic review and meta-analysis.

We searched PubMed, Embase, and the Cochrane Library database from January 1, 1993, to November 30, 2015, using both Medical Subject Heading (Mesh) terms and free text terms: ("breast cancer" OR "breast neoplasm") AND ("SLNB" OR "sentinel lymph node biopsy" OR "sentinel lymph node dissection") AND ("preoperative therapy" OR "preoperative chemotherapy" OR "neoadjuvant chemotherapy" OR "NAC"). Only articles published in English were selected. The search strategy is presented in Fig 1.

### Study inclusion criteria

Studies included in this review had to meet the following criteria: First, the patients had received NAC for invasive breast cancer. Second, the patients were clinically node-negative at the time of diagnosis. Clinically node-negative was defined as the absence of suspicious or abnormal-appearing lymph nodes on physical examination or ultrasound imaging. Third, the patients had undergone an SLNB after NAC, followed by an ALND. Patients receiving neoadjuvant endocrine therapy or preoperative radiotherapy were excluded from this meta-analysis.

### Study quality assessment

Two reviewers independently evaluated the study quality using QUADAS 2 [23], a standardized tool for the quality assessment of diagnostic accuracy studies. QUADAS 2 comprises four domains: patient selection, index testing, a reference standard, and flow and timing. The risk of bias is deemed "low," "high," or "unclear." If the answers to all signaling questions for a domain are "yes," then the risk of bias can be judged as low. If any question is answered "no," the risk of bias should be considered high. Applicability concerns were judged using similar criteria. The questions adopted in our review are listed in S1 File.

### Data extraction and definitions

The data were independently extracted by 2 reviewers and checked by other reviewers for accuracy. Discrepancies were resolved by consensus after discussion. The identification rate (IR) was defined as the number of women with successfully identified sentinel lymph nodes (SNs) divided by the total number of women for whom SLNB was attempted, using the histological analysis of the axillary lymph node collected with ALND as the "gold standard". The results of each successfully identified SN were further categorized as true positive (TP), true negative (TN), or false negative (FN). Four test performance parameters were evaluated: sensitivity [TP/(TN+FN)], false negative rate (FNR) [FN/ (FN+TP)], negative predictive value (NPV) [TN/ (TN+FN)] and accuracy rate (AR) [(TP+TN)/total number of successful SLNB].

### Statistical analysis

The meta-analysis in this study was conducted using R, version 3.2.2 for windows (R: A language and environment for statistical computing; R Foundation for Statistical Computing, Vienna, Austria, http://www.R-project.org/).

The meta-analyses of the IR, AR, FNR, NPV and sensitivity of SLNB were conducted using the *metaprop* function in the R-*meta* package. The individual studies were weighted by study size and by the inverse of the variance of the individual point estimates. The heterogeneity of the studies was evaluated using the inconsistency statistic (I^2^) [24]. Publication bias was displayed graphically using forest plots. The effect of lymph node mapping techniques on the IR and the use of immunohistochemistry (IHC) staining on FNR was determined using the chi-squared test. Two sided p-values<0.05 were considered significant.

## Results

### Description of included studies

Based on the search strategy, a total of 1,456 patients in 16 studies who met the inclusion criteria were analyzed [25–40]. QUADAS 2 was used to assess the quality of the studies included in our review. The results of the quality assessment are listed in Table 1. In the included studies, only the risk of bias in patient selection was considered high risk; the other aspects were low risk.

**Table 1:** Results of quality assessment according to QUADAS 2 for the included studies.

Study	Risk of bias				Applicability concerns		
	Patient selection	Index test	Reference standard	Flow and timing	Patient selection	Index test	Reference Standard
Nason et al.	2	1	1	1	1	1	1
Tafra et al.	2	1	1	1	1	1	1
Piato et al.	2	1	1	1	1	2	1
Tanaka et al.	2	1	1	1	1	2	1
Yu et al.	2	1	1	1	2	1	1
Kinoshita et al.	2	1	1	1	1	2	1
Gimbergues et al.	2	1	1	1	1	2	1
Papa et al.	2	1	1	1	1	2	1
Classe et al.	2	1	1	1	1	1	1
Hunt et al.	2	1	1	1	1	2	1
Dalus et al.	2	1	1	1	1	1	1
Pecha et al.	2	1	1	1	1	2	1
Aguirre et al.	2	1	1	1	1	2	1
Takahashi et al.	2	1	1	1	1	1	1
Madrona et al.	2	1	1	1	1	2	1
Kika et al.	2	1	1	1	1	2	1

1: low risk. 2: high risk.?: unclear risk

The 16 studies were published between 2000 and 2015. Fourteen studies were single-center studies [25, 27–32, 34–40], and the other two were multi-center studies [26, 33]. Four studies were from Japan [28, 30, 38, 40], 3 from the United States [25, 26, 34], 2 from France [31, 33], 2 from Spain [37, 39], and 1 each from Austria [35], Brazil [27], the Czech Republic [36], Israel [32], and Chinese Taipei [29]. All of the studies included a group of patients who were clinically node-negative at the time of diagnosis. All of these patients underwent SLNB and followed by ALND. Four studies performed SN mapping using radiocolloid alone [27, 31, 32, 37], three studies used blue dye alone [28, 29, 40], and 6 studies used both radiocolloid and blue dye [25, 26, 33–35, 38]. Regarding histological technique, 6 studies used hematoxylin and eosin (H&E) staining alone [27, 28, 30, 32, 37, 40], 3 studies performed additional IHC staining for negative nodes based on H&E staining results [25, 31, 33], and 3 studies used IHC staining in all cases [29, 34, 38]. Detailed descriptions of the included studies are summarized in Table 2.

**Table 2:** Detailed descriptions of included studies.

Study	Publication year	Origin	No. of patients SLNB attempts	Initial tumor size	Chemotherapy	SLNB mapping	IHC
Nason et al.	2000	USA	9	T1-4	AC	BD+RC	Yes
Tafra et al.	2001	USA	29	T1-2	m	BD+RC	Yes^a^
Piato et al.	2003	Brazil	42	T1-2	AC	RC only	No
Tanaka et al.	2005	Japan	17	T1-4	CPF	BD only	No
Yu et al.	2006	Chinese Taipei	127	T3	Doxorubicin based	BD only	Yes
Kinoshita et al.	2007	Japan	54	T2-4	FEC→T or T	BD/RC/Both	No
Gimbergues et al.	2008	France	82	T1-3	CAF or E→T or T	RC only	Yes
Papa et al.	2008	Israel	31	T2-3	AC	RC only	No
Classe et al.	2009	France	130	T0-3	m	BD+RC	Yes
Hunt et al.	2009	USA	575	T1-3	m	BD+RC	Yes^b^
Dalus et al.	2011	Austria	13	T1-4	Mainly epirubicin based	BD+RC	Yes
Pecha et al.	2011	Cezch	172	T1-4	Anthracycline based	RC only/BD only	Yes^c^
Aguirre et al.	2012	Spain	51	T1-3	AC→T	RC only	No
Takahashi et al.	2012	Japan	41	T1-4	Capecitabine +T→FEC/FEC→T/T	BD+RC	Yes
Madrona et al.	2015	Spain	49	T1-4	Mainly anthracycline and taxane contained	RC only/Both	m
Kika et al.	2015	Japan	34	T1-4	Mainly anthracycline and taxane contained	BD only	No

m, missing value; A, adriamycin(doxorubicin); C, cycolophosphamide; P, pirarubicin; F, 5 fluorouracil; E, epirubicin; T, taxol; BD, blue dye; RC, radiocolloid.

^a^ performed on majority of SN-negative patients for metastasis on H&E staining.

^b^ only performed on patients enrolled after 2000.

^c^ only performed on SN patients with suspicious malignant cells.

### Identification rate of SLNB

**Fig 2:**
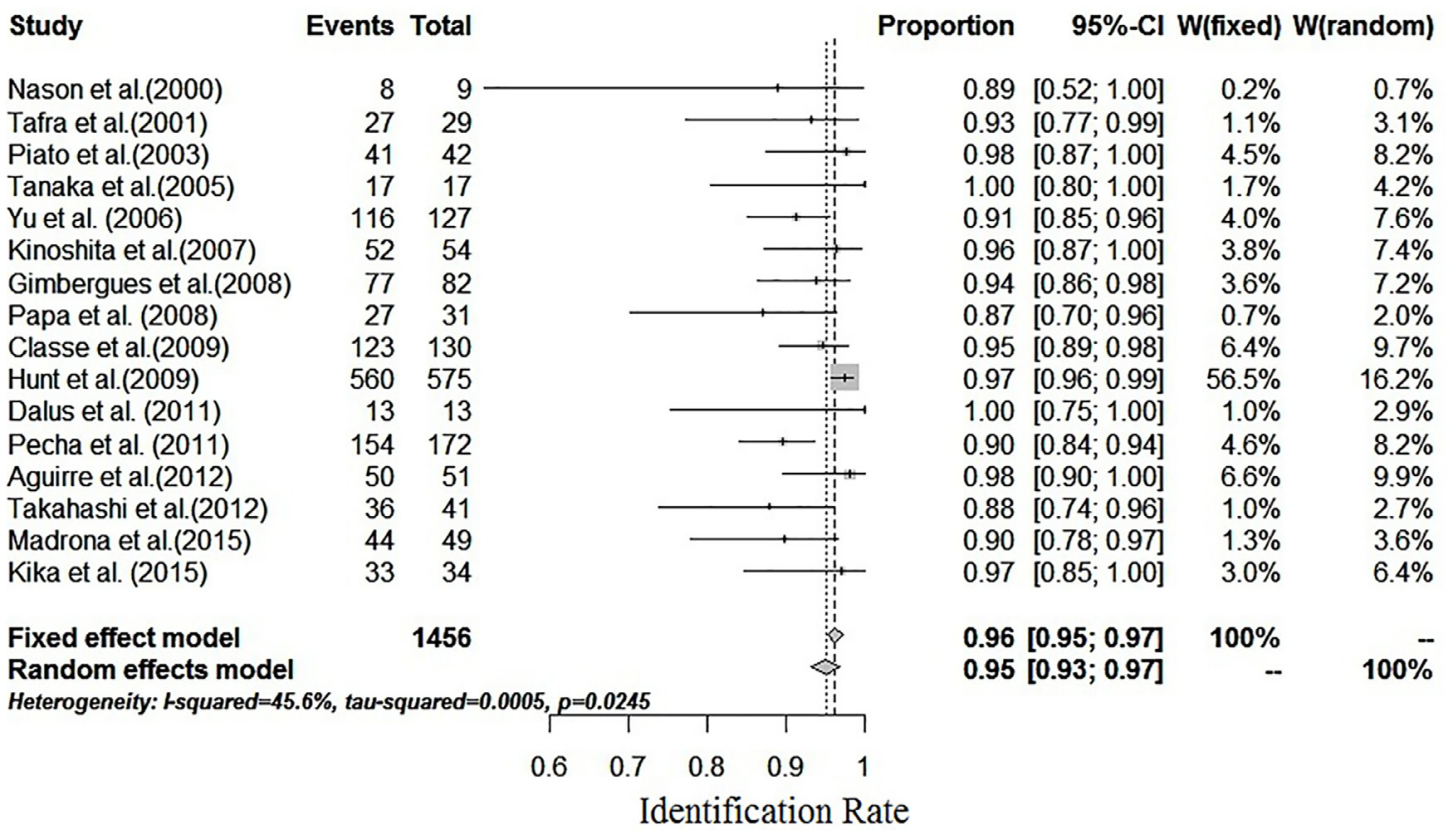
Forest plot of the IR. A fixed-effects model was used to estimate the combined IR, with a result of 96% (95% CI: 95%-97%); I^2^ = 45.6%.

**Fig 3:**
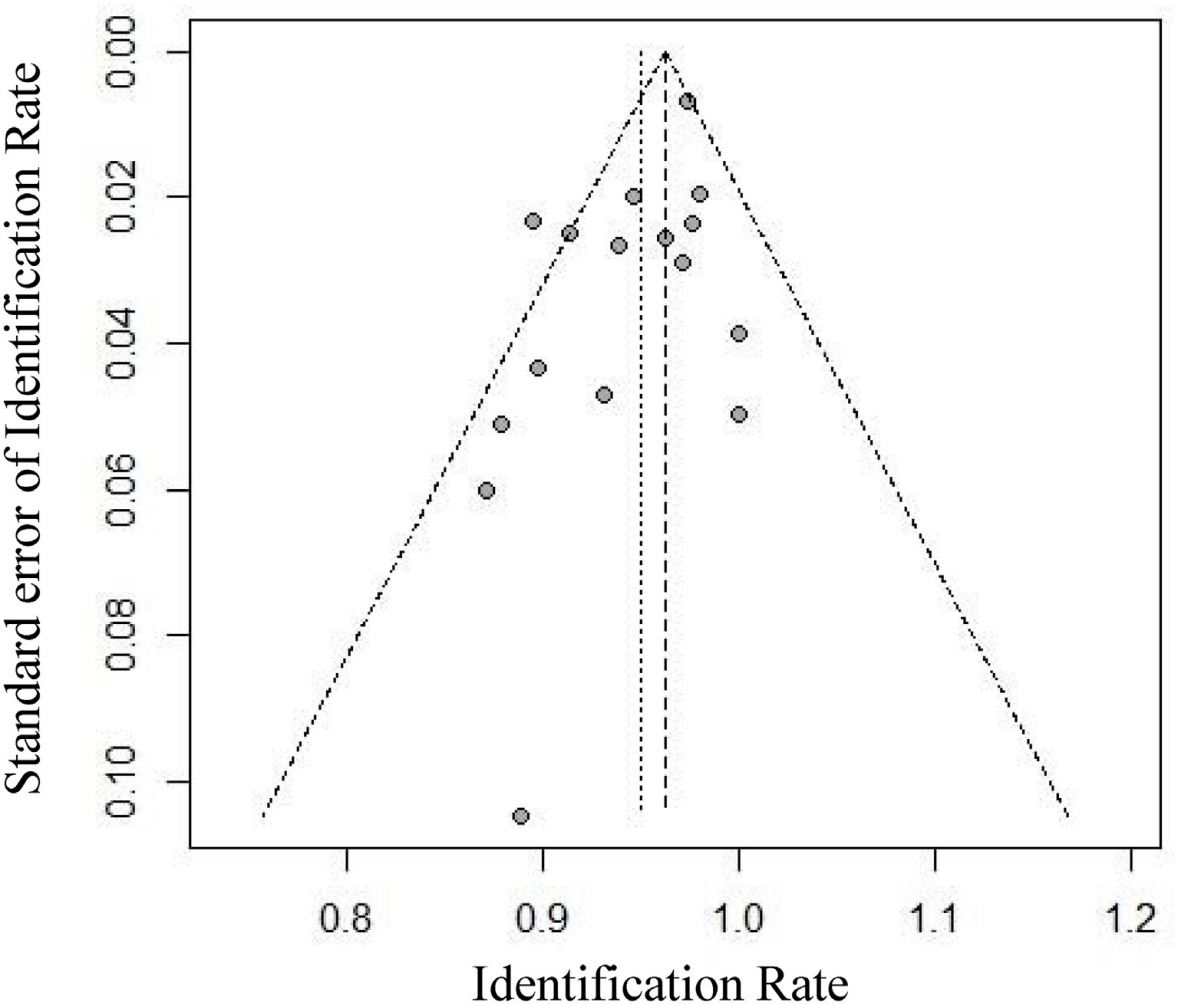
Funnel plot to assess the publication bias effect on the IR. Each dot represents a separate study. The funnel plot revealed no apparent evidence of publication bias.

In the 16 studies, the IRs of SLNB ranged from 87% to 100%. Moderate between-study heterogeneity (I^2^ = 45.6%, P = 0.0245) in the IR was found. Therefore, a fixed-effects model was used to estimate the combined IR with a result of 96% [95% confidence interval (CI): 95%-97%] (Fig 2). Funnel plots were used to assess the publication bias of all but one of the studies. The studies showed good symmetry and suggested minimal publication bias (Fig 3). Begg’s test confirmed the above conclusion, with P = 0.3219.

The mapping method was assumed to be associated with the IR of SLNB; therefore, we compared the IR rate according to the mapping method used. Three studies were excluded from this analysis because different mapping methods were used within a single study [30, 36, 39]. The summarized IR of the 3 studies that used only blue dye mapping was 96% (95% CI: 91%-100%). The pooled IR from the 4 studies that used only radiocolloid was 96% (95% CI: 94%-99%). The pooled IR from the 6 studies that used both blue dye and radiocolloid was 97% (95% CI: 96%-98%). There were no significant differences in the IR of SLNB among the different mapping methods (P = 0.180; Table 3).

**Table 3:** Identification rate of SLNB according to mapping method.

Mapping method	No. of studies	No. of patients SLNB attempts	No. of patients SN successfully identified	Identification rate (95% CI)
Blue dye alone	3	178	166	96% (91%-100%)
Radiocolloid alone	4	206	195	96% (94%-99%)
Blue dye + radiocolloid	6	797	767	97% (96%-98%)

Three studies were excluded from this analysis due to different mapping methods within a single study.

### False negative rate of SLNB

**Fig 4:**
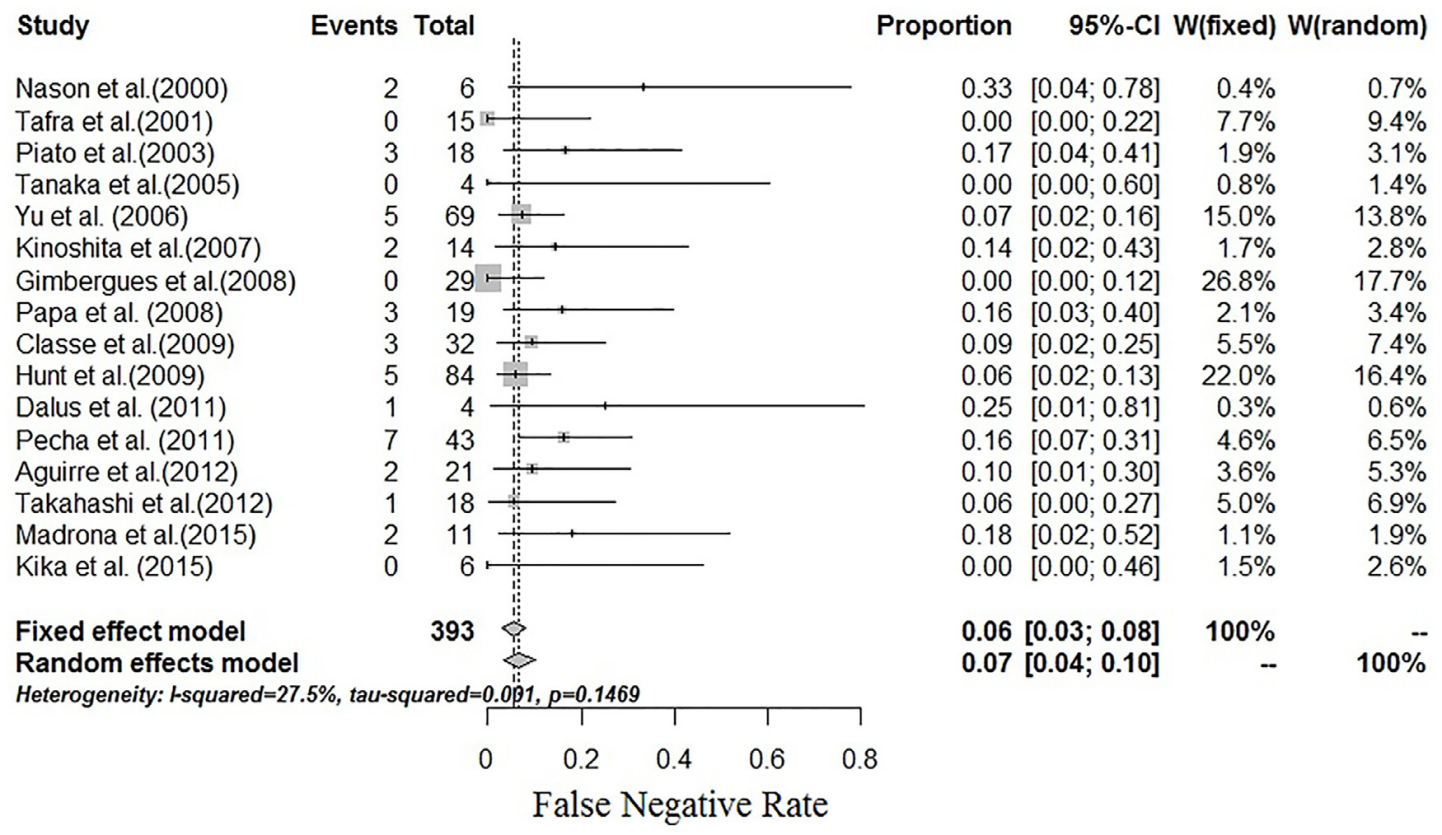
Forest plot of the FNR. A fixed-effects model was used to estimate the combined FNR with a result of 6% (95% CI: 3%-8%) I^2^ = 27.5%.

**Fig 5:**
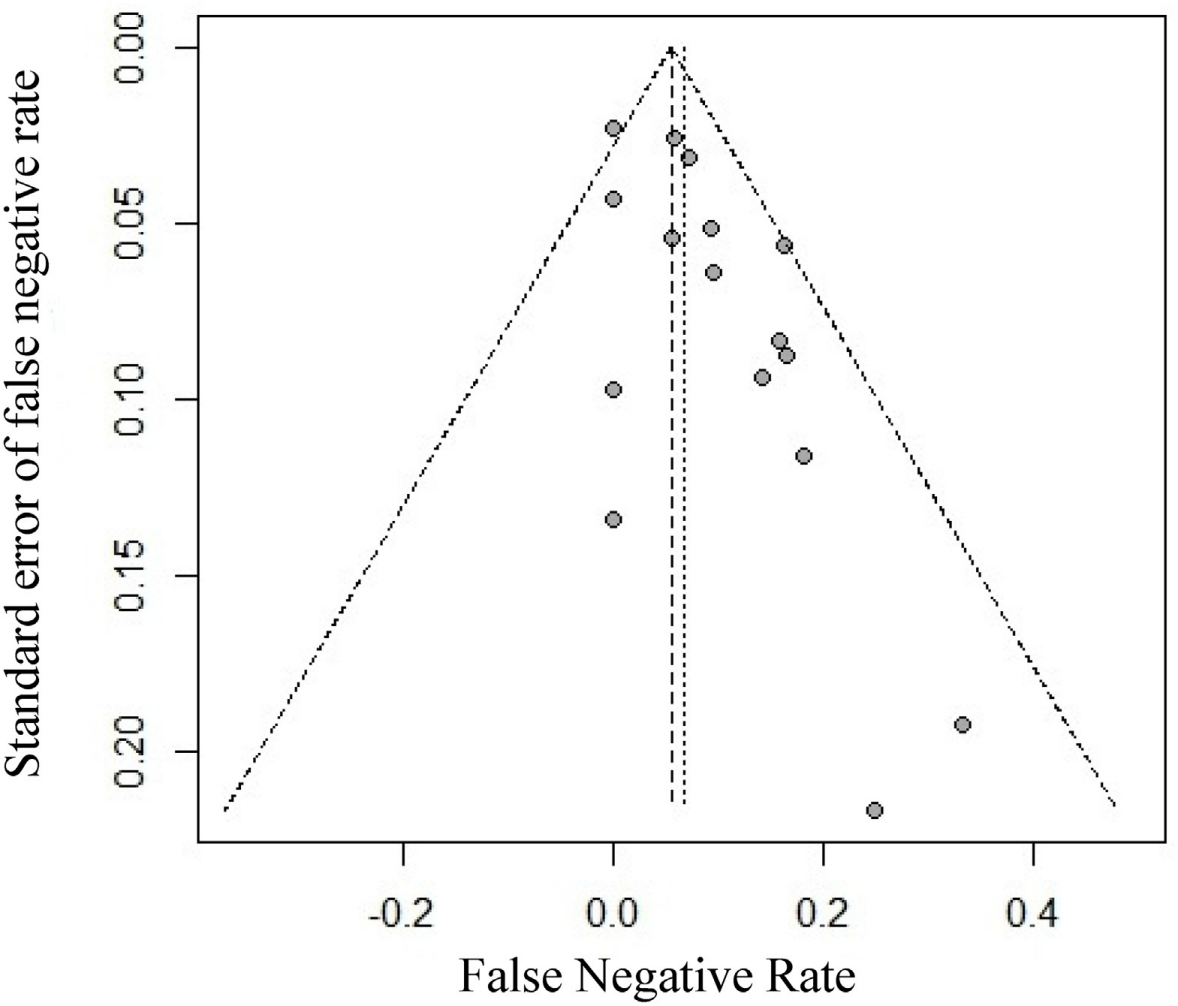
Funnel plot to assess publication bias effect on the FNR. Each dot represents a separate study. The funnel plot revealed no apparent evidence of publication bias.

The individual FNRs of SLNB ranged from 0% to 33% in the 16 studies. Moderate FNR heterogeneity was found among the studies (I^2^ = 27.5%, P = 0.1469; Fig 4). The summarized estimated FNR for the 16 studies was 6% (95% CI: 3%-8%) based on the fixed-effects model. The funnel plot showed minimal publication bias (Fig 5), and all studies used the funnel plot. This result was confirmed using Begg’s test, with P = 0.08711.

It is generally accepted that IHC staining with anti-cytokeratin antibodies is much more sensitive than conventional H&E staining for detecting the micrometastasis of lymph nodes [41, 42]. We compared the FNR rates of the different histological techniques that were used. Four studies were omitted because of incomplete information regarding the histological method [26, 34, 36, 39]. The pooled FNR from the 6 studies that only used H&E staining was 11% (95% CI: 4%-18%). The pooled FNR from the 6 studies that used H&E combined with IHC staining was 4% (95% CI: 1%-7%). No significant difference in the FNR of SLNB was detected between the studies that used IHC staining and those that did not (P = 0.241; Table 4).

**Table 4:** False negative rate of SLNB according to histological technique.

Histological technique	No. of studies	No. of patients with positive axillary lymph nodes	No. of patients with false negative SNs	False negative rate (95% CI)
IHC staining only	6	82	10	11% (4%-18%)
IHC staining combined with H&E staining	6	158	12	4% (1%-7%)

Four studies were omitted due to incomplete data regarding the histological method.

### Sensitivity, NPV and AR of SLNB

Three test performance parameters of SLNB were analyzed: sensitivity, NPV, and AR (Table 5). In the 16 studies, the sensitivity, NPV and AR ranged from 67%-100%, 50–100% and 75%-100%, respectively. The pooled sensitivity, NPV and AR were 94% (95% CI: 92%-97%, I^2^ = 27.5%), 98% (95% CI: 98%-99%, I^2^ = 42.7%) and 99% (95% CI: 99%-100%, I^2^ = 32.6%), respectively. The forest plots displayed minimal variation, and a fixed-effects model was used for the analysis.

**Table 5:** Sensitivity, NPV and AR in individual studies.

Study	Sensitivity (95% CI)	NPV (95% CI)	AR (95%)
Nason et al.	67% (22%-96%)	50% (7%-93%)	75% (35%-97%)
Tafra et al.	100% (78%-100%)	100% (74%-100%)	100% (87%-100%)
Piato et al.	83% (59%-96%)	88% (70%-98%)	93% (80%-98%)
Tanaka et al.	100% (40%-100%)	100% (75%-100%)	100% (80%-100%)
Yu et al.	93% (84%-98%)	91% (81%-97%)	100% (97%-100%)
Kinoshita et al.	86% (57%-98%)	95% (83%-99%)	96% (87%-100%)
Gimbergues et al.	100% (88%-100%)	100% (93%-100%)	100% (95%-100%)
Papa et al.	84% (60%-97%)	73% (39%-94%)	89% (71%-98%)
Classe et al.	91% (75%-99%)	97% (91%-99%)	98% (93%-99%)
Hunt et al.	94% (87%-98%)	99% (98%-100%)	99% (98%-100%)
Dalus et al.	75% (19%-99%)	90% (55%-100%)	92% (64%-100%)
Pecha et al.	84% (69%-93%)	94% (88%-98%)	95% (91%-98%)
Aguirre et al.	90% (70%-99%)	94% (79%-99%)	96% (86%-100%)
Takahashi et al.	94% (73%-100%)	95% (75%-100%)	100% (90%-100%)
Madrona et al.	82% (48%-98%)	94% (81%-99%)	95% (85%-99%)
Kika et al.	100% (54%-100%)	100% (87%-100%)	100% (89%-100%)
Pooled estimate	94% (92%-97%)	98% (98%-99%)	99% (99%-100%)

## Discussion

This present systematic review was conducted to provide an overview of the published literature regarding the feasibility and accuracy of SLNB in initially clinically node-negative breast cancer after NAC. We specifically evaluated the IRs and FNRs, which are the most important test performance parameters in SLNB. The pooled IR was 96%, with a 95% credible interval of 95 to 97%. The pooled FNR was 6%, with a 95% credible interval of 3 to 8%. These rates do not differ substantially from those reported in prior studies that accepted the use of SLNB in early breast cancer without NAC [43–45]. SLNB appears to be a reliable technique for staging initially clinically node-negative breast cancer after NAC.

Seven meta-analyses concerning SLNB after NAC have been performed. Three of them included both clinically node-positive and node-negative patients prior to NAC [46–48]. Although these 3 meta-analyses reviewed studies from different timespans, similar results were obtained, indicating that SLNB is a potentially reliable staging technique for breast cancer after NAC. Tan and his colleagues investigated the feasibility of SLNB in patients who were clinically node-negative after NAC [49]. The pooled IR and pooled FNR were 94% and 7%, respectively, and were comparable to the rates for SLNB without NAC. Two meta-analyses focused on SLNB in initially clinically node-positive patients after NAC [50, 51]. Both studies had acceptable IRs; however, the FNRs were poor (14% and 15.1%, respectively). Mocellin and his colleagues reviewed studies about SLNB after NAC in locally advanced breast cancer [52]. Their review included 72 studies and 7,451 patients. The IR was 89.6%, and the FNR was 14.2%. The performance of SLNB was not as good as that observed in early breast cancer. The current study is the first meta-analysis to investigate the feasibility and accuracy of SLNB in patients with clinically node-negative breast cancer after NAC. The IR found in this study was higher than that of previous meta-analyses, and the FNR was lower.

Currently, the standard treatment of the axilla after NAC is ALND. The reliability of SLNB in patients undergoing NAC remains controversial [18, 53]. Theoretically, chemotherapy could induce lymphovascular shrinkage and fibrosis, which may affect lymphatic mapping and lead to a lower IR. In addition, uneven sterilization of the lymph node tumor in NAC could lead to a higher FNR. These factors could explain why the IR and FNR of SLNB are not very good in patients after NAC. However, these possibilities have not yet been confirmed [31]. The situation seems quite different in early breast cancer. In Hunt’s study, which included 575 initially clinically node-negative patients with breast cancer who underwent SLNB after NAC, the IR was 97.4%, and the FNR was 5.9% [34]. Van der Heiden-van der Loo and his colleagues’ retrospective study showed similar IRs for SLNB in initially clinically node-negative patients before and after NAC (98% vs 95%) [54]. Santa-Maria et al. reported that there were no compelling data to support routine SLNB before NAC and recommended performing SLNB after NAC in patients with clinically node-negative axilla at presentation [53]. The latest ASCO guideline states that SLNB may be performed before or after NAC, but there is no convincing evidence supporting SLNB after NAC in patients with breast cancer [8]. In the present meta-analysis, we obtained a high IR, sensitivity, AR, and NPV and a low FNR for SLNB after NAC in initially clinically node-negative patients. These results directly support Santa-Maria’s opinion.

In initially clinically node-negative patients, avoiding additional surgical procedure before NAC could be the most important advantage of performing SLNB after NAC. To gain this advantage, two conditions are required: one is that SLNB could stage the axilla accurately after NAC, and the other is that SLNB could provide a safe alternative to ALND in SN-negative down-staged axilla. By pooling the results from these 16 studies, we demonstrate that SLNB is an accurate technique for axillary staging in this population. The first condition mentioned above was met. Kim et al.’s study performed SLNB after NAC in patients who had cytology-proven node-positive disease before NAC [55]. They found that in SN-negative patients, there was no significant difference in axillary recurrence between the SLNB-only and ALND groups. This result indicated that even when SLNB was performed after NAC, ALND was not necessary for SN-negative patients. The second condition was also met. Taken together, these findings indicate that performing SLNB and breast surgery in a single procedure after NAC is reasonable.

Initially clinically node-negative breast cancer patients have a relatively small tumor burden in the lymph nodes. NAC kills tumor cells in the lymph nodes, which can result in the downstaging of macrometastasis in SNs to micrometastasis. In this specific population of patients, macrometastases in SNs have a relatively high likelihood of shrinking to micrometastasis after NAC. Several studies have recommend to omitting an ALND in patients with micrometastasis and isolated tumor cells in SNs [56, 57]. If we regard SNs with micrometastasis after NAC as negative node, these patients could avoid an ALND. This could be another advantage of performing SLNB after NAC in initially clinically node-negative breast cancer patients. The SN FNAC study recommends that tumor of any size be considered positive in SLNB after NAC in biopsy-proven node-positive breast cancer [58]. In their study, if an SN with isolated tumor cells after NAC had been considered negative, the FNR would have increased to 13.3%. Whether ALND could be omitted in initially clinically node-negative patients with micrometastasis in SNs after NAC is unclear. More studies are needed to evaluate the clinical significant of micrometastasis in SNs after NAC in this specific patient population.

In our meta-analysis, the summarized IRs of the blue dye-alone group, the radiocolloid-alone group and the combined group were 96%, 96% and 97%, respectively. No significant difference was detected with the chi-square test. The different mapping methods did not influence the IRs. Boughey and his colleagues evaluated the factors affecting SN identification after NAC [59]. The IRs of SLNB were 78.6% with blue dye alone, 91.4% with radiocolloid and 93.8% with dual mapping agents. The researchers concluded that the IR of SLNB after NAC was higher when mapping was performed using radiocolloid either alone or combined with blue dye than with blue dye alone. The different results that we obtained may be related to the fact that the initial axillary status varied among the patients included in our systematic review.

Opinions differ on the influence of IHC on the FNR of SLNB after NAC. In Tan’s review, the summary FNRs of SLNB after NAC were 9% and 12% in the groups with and without IHC utility, respectively (P = 0.45) [49]. Meanwhile, a meta-regression conducted by Fu et al. identified IHC as an independent factor underlying the heterogeneity of FNR (P = 0.04) [50]. Further analysis revealed that the FNR in the IHC-plus-H&E group was significant lower than in the H&E-alone group (8.7% vs 16.0%, P = 0.001). In the present meta-analysis, the pooled FNRs were 4% in the IHC-plus-H&E group and 11% in the H&E-alone group. There was no significant difference between the two groups (P = 0.241). Further studies are needed to investigate the benefit of IHC in SLNB after NAC.

It has been reported that in 40 to 70% of patients, the SN is the only involved axillary node in early breast cancer [60–62]. Predicting the status of non-SN will spare some SN-positive patients from ALND. The situation in initially clinically node-negative breast cancer patients after NAC is unclear. Evaluating the metastatic rate to non-SNs according to SN status in these patients would be of great value. We could only extract these data from 3 articles for analysis. The metastatic rate to non-SNs according to SN status is shown in Table 6. In Yu’s study, the metastatic rate in SN-positive patients is noticeably higher because only T3 tumor patients were included in that study. The information that we could obtain from those three studies was limited, and further study is needed to address this issue.

**Table 6:** The metastatic rate to non-SNs according to SN status in initially clinically node-negative breast cancer patients after NAC.

Study	Non SN metastatic rate in SN positive patients	Non SN metastatic rate in SN negative patients
Tanaka et al	0%(0/4)	0%(0/13)
Yu et al.	62.5%(40/64)	9.6%(5/52)
Kinoshita et al.	33.3%(4/12)	5%(2/40)

Only studies published in English were included in our meta-analysis, which may have led to publication bias. In addition, studies favoring the use of SLNB after NAC are more likely to be published, which may cause a bias in favor of SLNB. Funnel plots were used to evaluate the publication bias in the present meta-analysis, and the results indicated there was minimal publication bias. Begg’s test further confirmed these results.

In conclusion, based on current evidence, SLNB is technically feasible and accurate enough for staging the axilla in initially clinically node-negative breast cancer after NAC.

## Supporting Information

S1 File Questions used to assess the quality of the literature. (DOCX)

S1 Table PRISMA Checklist. (DOC)
