# Supplementary material for: The Feasibility and Accuracy of Sentinel Lymph Node Biopsy in Initially Clinically Node-Negative Breast Cancer after Neoadjuvant Chemotherapy: A Systematic Review and Meta-Analysis
Source: PLoS One. 2016 Sep 8;11(9):e0162605. doi: 10.1371/journal.pone.0162605 (PMC5015960; doi:10.1371/journal.pone.0162605)
Supplement: S1 File — (DOCX) [file pone.0162605.s001.docx]

The following signaling questions were used to assess the quality of the literature:

1 Patient selection

1.1 Risk of bias

1.1.1 Was a consecutive or random sample of patients enrolled?

1.1.2 Were patients who did not achieve a clinically complete response of the axillary node after NAC included?

1.2 Applicability concerns

Was the axillary node negative status evaluated by physical examination and Ultrasound?

2 Index test

2.1 Risk of bias

2.1.1 Were the index test results interpreted without knowledge of the results of the reference standard?

2.1.2 If a threshold was used, was it pre-specified?

2.2 Applicability concern

2.2.1 Were micro-metastases (<2 mm metastases) in the node considered positive in patients for whom IHC was performed?

2.2.2 Was blue dye combined with raidiocolloid were used as a mapping technique?

3 Reference standard

3.1 Risk of bias

3.1.1 Was the reference standard likely to correctly classify the target conditions?

3.1.2 Were the reference standard results interpreted without knowledge of the results of the index test?

3.2 Applicability concern

Did the reference standard match the question?

4. Flow and timing

4.1 Did all patients receive ALND?

4.2 Was the IR of the study higher than 90%?

4.3 Was the same reference standard used?

4.4 Was there an appropriate interval between the index tests and reference standard?
